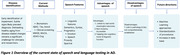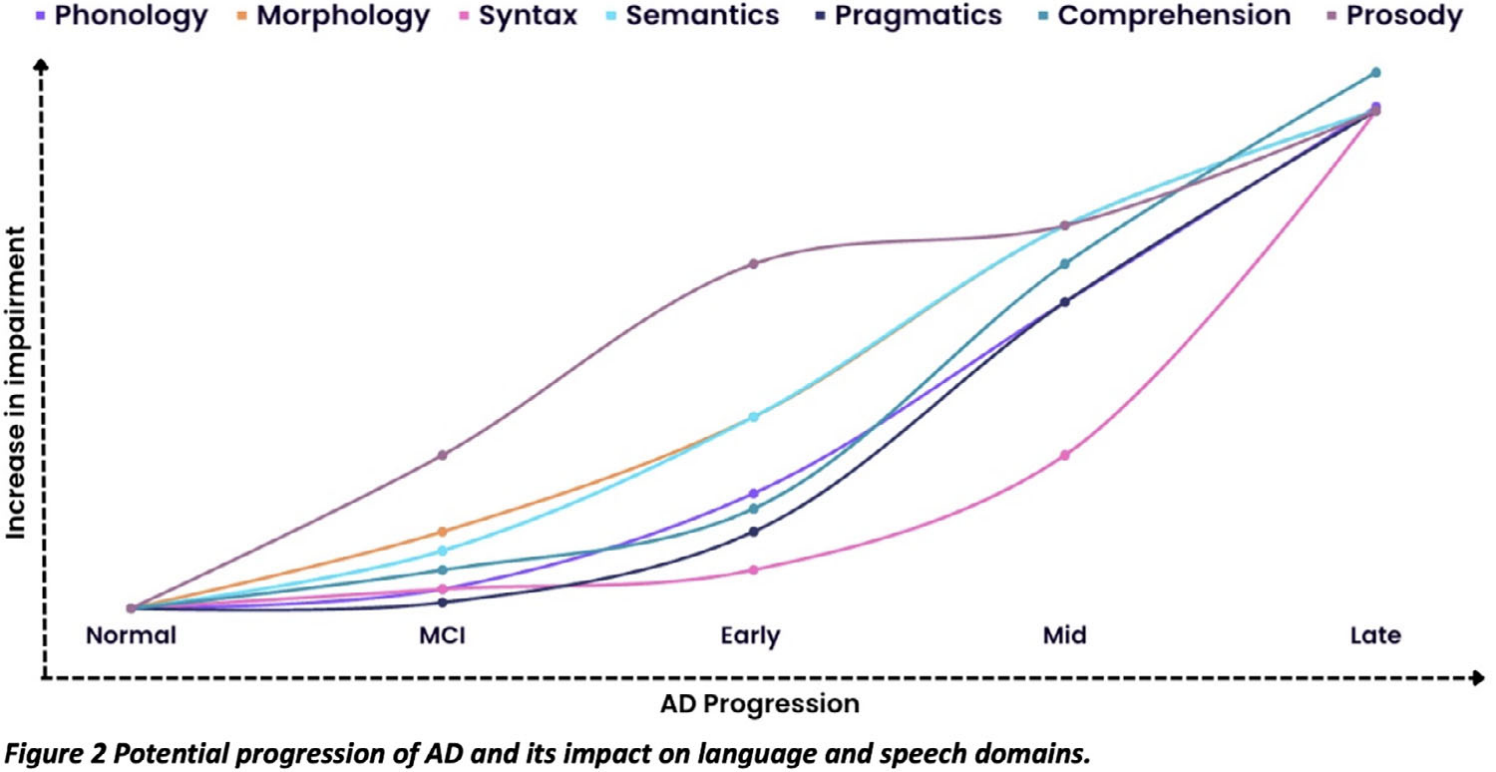# Update on the current state of speech and language testing in Alzheimer's Disease

**DOI:** 10.1002/alz.090397

**Published:** 2025-01-09

**Authors:** Shloka Dhareshwar, Ruby Mineur, Courtney Lewis, Melissa Kane, Paul Maruff, Adam P Vogel

**Affiliations:** ^1^ University of Melbourne, Melbourne, VIC Australia; ^2^ Cardiff University, Cardiff, Wales United Kingdom; ^3^ The University of Melbourne, Parkville, VIC Australia; ^4^ CogState, Melbourne, VIC Australia; ^5^ Florey Institute of Neuroscience and Mental Health, Parkville, VIC Australia; ^6^ The University of Melbourne, Melbourne Australia; ^7^ Redenlab, Melbourne, VIC Australia

## Abstract

**Background:**

Screening and disease monitoring are two core challenges of disease management in Alzheimer's Disease (AD). Digital speech and language features have shown promise as clinical outcomes in related disorders.

**Methods:**

We reviewed how speech digital markers are currently used in AD, focusing on how behaviours are connected to underlying disease pathology.

**Result:**

The rate of cognitive decline can be predicted by poor performance on language tests. The capacity of current language tests to distinguish between those with mild cognitive impairment (MCI) and healthy controls is limited. In some cases, disease progression can only be detected when significant changes in severity occur. Poor standardisation and limited clinical data are currently restricting communication outcomes uptake in clinical trials. The potential advantages of speech analytics and mobile health technology lie in their cost‐effectiveness, accessibility (non‐invasiveness), and automated analytical protocols. They have the capacity to improve assessment procedures, enhance screening and provide meaningful outcomes in clinical settings.

**Conclusion:**

High‐dimensional data can be collected by automatically and objectively analysing speech. Developments in machine learning, signal processing and natural language processing techniques will further optimise outcomes.